# Poultry Value Chain in Two Medium-Sized Cities in Kenya; Insights From Cluster Theory

**DOI:** 10.3389/fvets.2022.601299

**Published:** 2022-04-29

**Authors:** Samuel Onyango Omondi

**Affiliations:** ^1^Department of Agricultural Economics, University of Nairobi, Nairobi, Kenya; ^2^Department of Human Geography, Lund University, Lund, Sweden

**Keywords:** urban poultry farming, value chain governance, cluster theory, illegalities in poultry value chain, Thika, Kisumu, Kenya

## Abstract

Poultry is an attractive enterprise among urban smallholder farming households and is the most common livestock reared for home consumption and sale. By combining cluster and value chain approaches, the study analyses the operation of poultry value chain in medium-sized cities of Thika and Kisumu, Kenya. The study draws on a survey of 312 urban poultry producing households as well as qualitative interviews with key stakeholders in the urban poultry value chain. Spot market is the predominant governance structure in the poultry value chain in Thika and Kisumu. Farmers and traders employ various upgrading mechanisms to maintain their competitiveness. However, some producers and traders engage in illegal activities such as theft of poultry, illegal slaughtering, and sale of adulterated low-quality poultry feed. Results also show that poultry producers in Thika enjoy the benefits of being located in a cluster of feed millers and close proximity to output market.

## Introduction

Farming within and around cities has existed since time immemorial (1). Urban agriculture is today practiced by households of all income groups and is an important livelihood strategy among poor urban households (2). An array of factors contributes to the development of urban agriculture in developing countries. On one hand, there are push factors including rising unemployment rates and widespread urban poverty and on the other hand, there are pull factors including rising demand for urban agriculture products and close proximity to input and output markets (3).

In developing countries, urbanization, rapid urban population growth, and improvement in welfare have resulted in a dietary transformation, characterized by a shift from consumption of mainly cereals to a diet with more meat, dairy products, fruits, and vegetables. Forming a part of this dietary change, the growing demand for animal protein presents opportunities for growth of the meat industry (4). Global meat production is projected to grow by about 14 per cent by 2030 compared to the base period of 2018–2021, with the rise in demand for poultry meat being a major driver for the change (5). The most widely culturally acceptable animal protein is poultry meat and eggs. Poultry meat will constitute 41 per cent of all meat consumed globally by 2030 (5). In Kenya, poultry meat consumption is expected to increase from 55 thousand metric tons in 2000 to 165 thousand metric tons in 2030 (6).

Agriculture is the backbone of Kenya's economy, contributing one third to the Gross Domestic Product (GDP) in 2015 (7). It's one of the six sectors of the economy, identified in the country's Vision 2030, with the potential of driving economic growth (8). The Ministry of Agriculture, Livestock and Fisheries, through its strategic plan of 2013–2017, aimed at promoting urban and peri-urban agriculture to address the effects of urbanization on food security (9). Although there is an increased interest in urban agriculture from both researchers and development agencies (10–12), the focus has mainly been on crop production, particularly horticulture and to a lesser extent on dairy production (13–15). Small livestock production in urban areas has received far less attention, especially the not readily visible small animals reared in the backyards, such as poultry. Though mostly given a brief mention in urban agriculture studies, poultry tops the list as the most common livestock reared in African towns (16–19).

Poultry is an umbrella term for various types of domesticated birds including chicken, ducks, geese, turkey, guinea fowls, quails, and pigeons. One reason for the attractiveness of poultry production in developing countries is its relatively smaller space requirement compared with other kinds of livestock and staple crop production (20). Poultry enterprises also require relatively lower capital and the returns are usually quicker than in other livestock enterprises. Indeed, chicken production in Kenya increased from 44 million heads in 2016 to about 57 million heads in 2020 (21). Indigenous chicken makes up the lion share of 84 per cent, with the rest being layers (8%), broilers (6%), and other poultry species (2%) (8). In Kenya, poultry production performs several livelihood roles at the household level: it provides food in form of meat and eggs, income from sale of meat, eggs and manure and acts as capital for investments (22).

Studies show that producers of high value agricultural commodities such as horticulture and livestock products are occasionally integrated into the various value chains through contract farming (23). For example, in the poultry subsector, broiler production can be vertically integrated with a lead firm providing farmers with production inputs while farmers provide housing and management for the birds. The lead firm provides market for broilers and compensates farmers for the housing and management of birds (23). Other farmers produce and market broilers independently (24).The referred to studies mainly, if not totally, focus on rural producers. While these practices are characteristic for rural agricultural production, the question is, to what extent do they apply in urban agricultural production?

The value chain approach sheds more light on the operations of an industry than a narrow focus on only one or a few firms within an industry. This is because the inefficiencies as well as value added could be situated at nodes other than those being focused on by studies addressing one of the nodes of the value chain. Value chain analysis transcends different sectors allowing for a wider look at an industry (25). However, as Porter (26) argues, “competitive advantage is created and sustained through a highly localized process” (p. 73). Porter emphasizes the role of firms clustering on competitiveness and innovation. While it is advantageous to have a broader birds' eye view of a sector through value chain perspective, cluster theory provides more insights on local level factors and interactions among value chain actors that enhance competitiveness in an industry. Value chain and cluster theory have further been discussed in Section Theoretical Framework.

Thus, this study aims at answering three questions; How does the contextualization of poultry value chain in medium-sized towns enhance our understanding of how it operates?; What is the governance structure and institutional framework of the urban poultry value chain?; How does the application of cluster theory to value chain improve our understanding of urban poultry value chains? In addition, the study aimed at characterizing the urban poultry value chain by mapping the prevalence of the different poultry production ventures.

## Theoretical Framework

This section presents the theoretical underpinnings of the value chain approach and cluster theory. While value chain approach provides a broad eye view of the poultry sector and the different upgrading mechanisms, cluster theory provides insights why poultry producers and input suppliers cluster in certain locations. The section ends by presenting application of cluster theory in value chain analysis.

### Value Chain Approach

A value chain entails all activities a firm or producer performs in designing, producing, delivering, and supporting its products, that is, activities ranging from product design, delivery of final product to consumers, and final disposal (25). As such, value chains focus on production, value addition, innovativeness, and marketing of products (27). It is this identification of the different components of a product chain that improves the understanding of its structure and functioning (28, 29). By doing this, value chain analysis may provide solutions for inefficiencies identified within a given chain and how the position of an actor can be upgraded. Furthermore, identification of areas of competitive advantage enables a firm to outsource functions which it is inefficient in carrying out [(25), p. 66].

The study of governance helps in transforming a value chain from a simple descriptive tool to an analytical instrument (25). Gereffi et al. [(30), p. 4] define value chain governance as the “non-market coordination of economic activities.” Issues to consider about governance revolve around power and institutional framework. Power can be expressed in two different ways; coercing other players into certain actions and activities; and “the capacity to be deaf to the demands of others” [(25), p. 66]. Large firms also tend to have more power through their known brand names, control of key technology, equipment, competences, political influence, and having large value added. In a poultry value chain, power could be expressed through possession of key knowledge or technical expertise in poultry production and dominance in the supply of inputs such as Day Old Chicks (DOCs) (31). Value chain maps plot major players within the chain whose behaviors determine success or failure of actors of interest.

According to Gereffi (32), global value chains have four dimensions; value added chain, geographic location of production and marketing, governance and power structures, and institutional framework. The value-added chain links products and resources to various actors while the location dimension maps the network of various actors both locally and globally. Governance entails authority and power which determines the flow of resources. The institutional framework which affects the operations of a value chain operates at the local, national, and international level. The current study conceptualizes the urban poultry value chain using three of the four dimensions of value chains, i.e., value chain governance and powers of actors, geographical location of major activities, and institutional framing of the value chain.

Gereffi et al. (33) describes five forms of governance in commodity value chains; markets, modular value chains, relational value chains, captive value chains, and hierarchy. Under market governance, there are often low costs to both parties for switching to other new partners, because transactions are not complex and there is low asset specificity. This is a characteristic of spot market exchange. Modular value chains involve suppliers of commodities customized to customers' specifications, while relational value chains are characterized by mutual dependence between buyers and sellers, as well as high asset specificity (33).

In captive value chains, small suppliers are dependent on large buyers, with high levels of monitoring and control by large firms (33). Poor coordination of supply in captive value chains could lead to oversupply of the commodity. Lastly, the hierarchy form of governance is typically a form of vertical integration, in which a firm performs several roles that would have otherwise been done by other firms (33). For example, a firm could deal with production of poultry, processing, and marketing as well as producing their own feed. These governance forms occur in a continuum, with markets and hierarchy (firm) at far ends (34).

The urban poultry value chain overlaps to global value chains through importation of grandparent stock and import and export of meat and eggs, hatching eggs, and raw materials for feed formulation (31, 35). However, this study focuses on the local, the cities and national level.

### Cluster Theory

In this study, a cluster is considered to be a group of related firms and industries located in close proximity to each other because of their commonalities and complementarities and they can either compete, cooperate, or both (36). They include the production firms, specialized input suppliers and service providers and supporting institutions such as universities, Non-Government Organizations (NGOs), farmer groups and trade associations. An industry could belong in more than one cluster through linkages to other sectors (26), for example, poultry industry is linked to tourism through demand created by hotels and restaurants, and, at the same time, linked to construction industry which provides the raw materials for construction of poultry houses.

The reasons for firms clustering could be grouped into two broad categories; demand and supply factors. On the demand side, firms cluster to benefit from a wide local demand for their products (37). Other firms locate to areas with related firms to gain a market share. Customers also provide essential information for innovation and therefore, most firms will locate in areas with large markets. Additionally, firms are purported to benefit from local rivalry which forces them to innovate to remain competitive (36). The three supply factors which provide clustering benefits or positive externalities date back to Marshall (38). First, firms concentrate in a given location to benefit from pooled specialized labor which improves their access to labor and contributes to employment. Secondly, firms concentrate in an area because they have access to specialized related inputs. The inputs are usually cheaper and in a wide variety. Thirdly, firms in a cluster benefit from knowledge spillovers, where knowledge is easily transferred to related nearby firms.

Empirical findings show that firms choose location based on knowledge spillovers and that they strategically locate to maximize the benefits of knowledge spillovers (39). This is because knowledge spillovers are necessary for innovation (40). Innovations or upgrading imply new production process or design, new marketing approach, or a new way of conducting training (26). Thus, the competitiveness of firms within an industry is contingent on the firms' innovativeness and ability to upgrade (26). The benefits of clustering further lead to growth of industries and emergence of new industries within or near clusters (41, 42). Furthermore, firms in a cluster are more likely to benefit from government grants for research and development than those not located in clusters (43).

### Applying Insights From Cluster Theory in Value Chain Analysis

While value chain analysis is concerned with the operation and organization of production and distribution of products, computation of value added, governance, and mapping value chain actors, cluster theory provides insights on how local interactions between actors in a value chain and local factors such as ease of access to specialized inputs and domestic demand shape the operation of the value chain (44). Merging the two approaches in the context of urban poultry production provides far much greater insights than using only either of the perspectives alone and others have applied the combination previously, for example Riedel et al. (45). For instance, how do urban poultry producers upgrade in the context of competition? Does the competition at the local level lead some actors to pursue illegal activities to remain competitive? How do local factors influence the conduct of value chain actors? Does concentration of closely related firms offer advantages to the firms? Cluster theory provides answers to these questions, which enrich the understanding of the performance of the value chain and actions of value chain actors.

## Methods

### Study Sites

This study was part of an urban agriculture research project in Kenya, Uganda and Ghana. It was part of a broad research titled ‘Urban Agriculture in African Cities'. The universities involved in the research include: Lund University, Swedish University of Agricultural Sciences, University of Nairobi (Kenya), Makerere University (Uganda), and University of Ghana. Kisumu City and Thika town were chosen in Kenya. The choice of cities in the study was purposive and based on practicality, contacts and understanding of urban food environment. Thus, all the respondents interviewed during the household survey were from the urban and peri-urban areas of the two cities.

Thika town, with a population of about 150,000 inhabitants is located in Kiambu County, about 50 km North of Nairobi (19, 46). Thika has a cluster of commercial poultry producers, feed companies and other related industries. Close to a third of all feed millers in Kenya are located in Thika or nearby Nairobi (47). The egg market in Kiambu County is well-established and the region exports eggs to the rest of the country (24, 35, 47). Kiambu County borders Nairobi, a major destination for agricultural commodities, which provides agricultural firms in Kiambu with a wide domestic market, one of the drivers of clustering. Furthermore, location within a cluster of related industries and firms reduces transaction costs and costs of inputs.

Kisumu City is located at the shores of Lake Victoria, in the Western part of Kenya. It covers three sub-counties; Kisumu Central, Kisumu East and some wards of Kisumu West, with a joint population of approximately half a million people (48). Although urban agriculture has a potential to provide livelihood to Kisumu residents, this opportunity has hitherto been neglected by the local authorities. The City has continued to rely on food imports from other counties (49). Compared to Thika, Kisumu has a lower number of feed millers.

### Sampling and Data Collection

The survey data in this study were collected in 2016, when interviews were carried out with 312 poultry farming households residing in the same communities that were surveyed in 2013 [for a comprehensive sampling procedure used in the urban agriculture baseline survey, see Omondi et al. (19)]. A list of all poultry farmers from the urban agriculture baseline survey of 2013 was used as the sampling frame. MS Excel random number generator was used to sample 135 and 177 farmers in Thika and Kisumu, respectively. However, because of difficulties in locating most farmers, only 45 of the sampled farmers were among those interviewed in 2013. Since the aim was to sample farmers from areas where baseline survey was conducted, missing farmers were replaced with their neighbors who reared poultry. Qualitative data were collected through four Focus Group Discussions (FGDs) with poultry farmers and Key Informant Interviews (KIIs) with poultry product traders and producers, input suppliers, hotel procurement managers, county livestock production officials, Non-Governmental Organizations (NGOs) representatives and poultry farmer groups, county staff, and through participant observations between July 2016 and October 2016. Due to sensitivity of some issues discussed, the list of participants in the FGDs and KIIs will not be presented. Besides these methods, the author also spoke at length with urban farmers, poultry products traders, and input dealers during unstructured tours at the farms and markets.

### Data Description and Analysis

The household survey enquired about the type of poultry and the number of poultry raised, marketing channels, input and output prices, farm, socio-economic, and demographic characteristics. Additionally, GPS (Global Positioning System) coordinates of location of poultry production were collected and mapped with reference to the main input and output markets for both cities. FGDs and KIIs elicited information on value chain governance, input acquisition, marketing, relationships with suppliers and behavior of producers and traders, including illegal activities.

Qualitative data from FGDs and KIIs were analyzed by grouping responses into themes. Data obtained from households were analyzed using SPSS. The software was used to compute means, frequencies and compare means using *t*-test.

## Results

### Insights From Value Chain Analysis

#### Main Actors in the Urban Poultry Value Chain, Production and Marketing Characteristics

**Figure 1:**
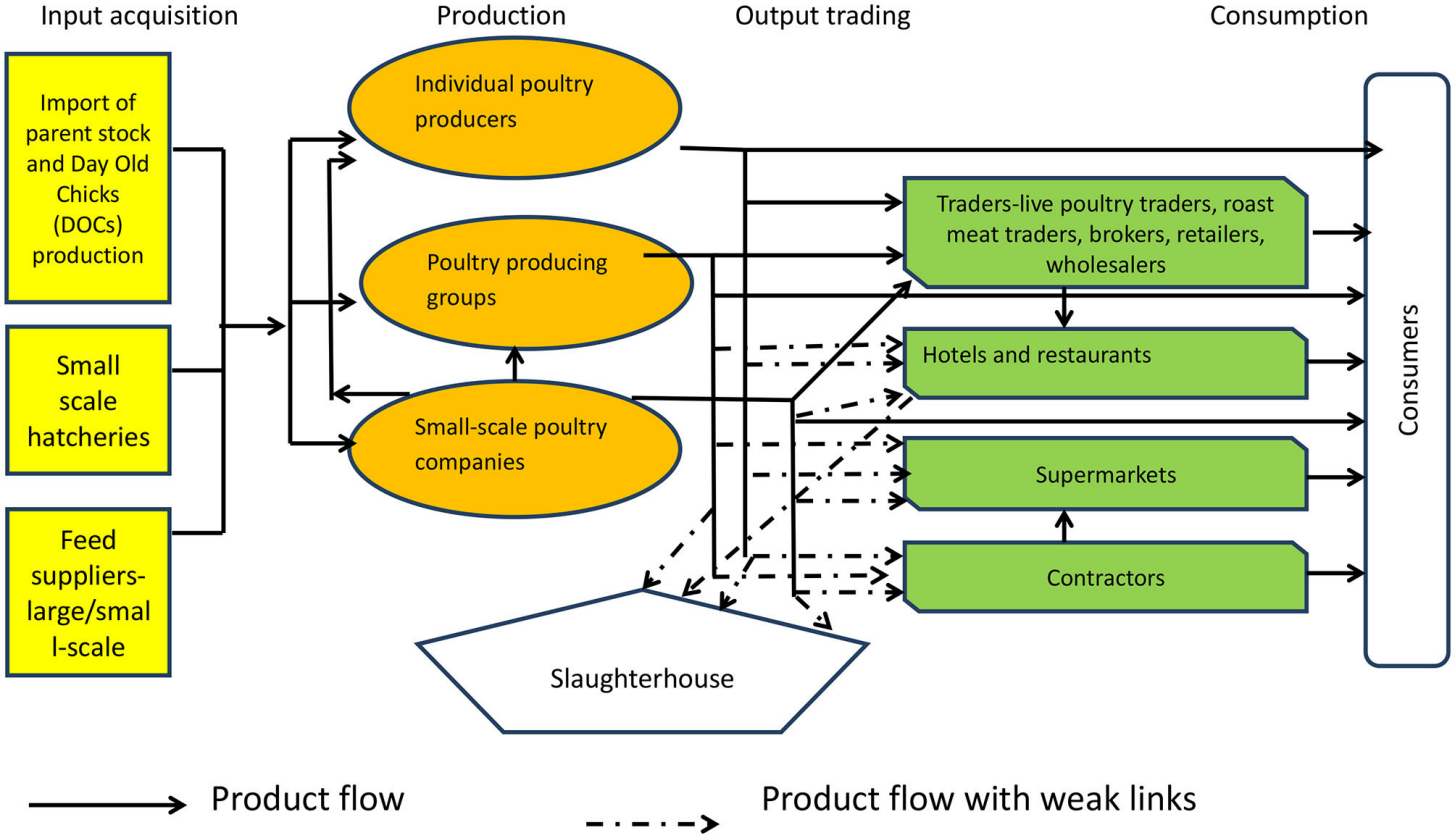
Indigenous chicken value chain in Thika and Kisumu, 2016.

One dimension of value chain analysis is mapping of value chain actors. The poultry value chain in Thika and Kisumu has an array of actors, who can broadly be categorized into five groups; input suppliers, producers, output traders, service providers, and consumers. The value chain map is presented in Figure 1. Importation of live poultry and fertile hatching eggs is regulated by the Directorate of Veterinary Services (DVS). DVS issues permits per consignment, accompanied by a Veterinary Health Certificate from country of origin to ascertain that animals are healthy (50). As from July 1 2021, Kenya introduced a 25 per cent excise duty (through the Finance Act, 2021) on imported fertilized eggs (51). Although the excise duty aims at protecting local farmers from low quality imports, it has made it expensive for hatcheries to import fertilized eggs. The implication is that the cost of day old chicks will trickle down to farmers and eventually to consumers, increasing the final output price.

What follows are descriptions of some of the actors' characteristics and their roles.

##### Individual Poultry Farmers Type, Prevalence and Number of Different Poultry Types Reared in Thika and Kisumu

About one out of five urban residents in Kisumu and Thika rear poultry, and of the urban farmers in the two cities, three fifths are keeping poultry (19). Table 1 presents the prevalence of different kinds of poultry kept and their numbers. Indigenous chicken, the most common type of poultry reared, are kept by four fifths of farmers with an average of 43 birds per farm. The proportion of farmers keeping indigenous chicken is higher in Kisumu than in Thika. Only 11 per cent of indigenous chicken farmers reported to keep improved chickens such as *Kuroiler*, KARI *Kienyeji*, and *Kenbro*. Ducks are the second most common poultry type in the surveyed areas, with close to a fifth of farmers rearing a small number of ducks (Table 1). Ducks are particularly common in low-income residential areas where they range freely, feeding in dump sites and waste water drainages. Prevalence of duck rearing in Kisumu is more than double that in Thika, probably because of the high number of informal settlements in Kisumu in which duck rearing is more practical.

**Table 1:** Percentage of farmers in Thika and Kisumu engaged in different poultry enterprises and the mean number of poultry kept, 2016.

**Enterprise**	**Whole sample**		**Kisumu**		**Thika**	
	**Share (%) (*N* = 312)**	**Mean number of birds**	**Share (%) (*n* = 177)**	**Mean number of birds**	**Share (%) (*n* = 135)**	**Mean number of birds**
Indigenous chicken	81.6	42.8 (62.9)	84.7	43.6 (54.4)	77.0	41.7 (73.8)
Layer	11.2	401.7 (293.6)	5.6	117.2 (88.6)	18.5	515.5 (268.2)^***^
Broiler	10.6	336.0 (295.4)	11.3	382.5 (279.7)	9.6	264.5 (315.7)
Duck	16.7	24.8 (21.8)	23.2	26.3 (22.0)	8.1	19.0 (20.8)
Turkey	2.2	19.6 (27.1)	2.8	25.2 (31.0)	1.5	5.5 (3.5)
Guinea fowl	2.2	21.0 (25.3)	2.8	26.8 (28.4)	1.5	6.5 (3.5)
Quails	0.3	500.0	0	0	0.7	500
Pigeons	1.3	21.5 (16.2)	1.1	8.0 (2.8)	1.5	(7.1)

*Figure in brackets is the standard deviation*.

^**^Significant at 5%,

*** *significant at 1% (comparison of mean was tested using t-test)*.

*Source: Author's survey, 2016*.

About 10 per cent of farmers produce both layers and broilers. The prevalence of layer production is three times that in Kisumu. Additionally, the average number of layers is significantly higher in Thika than Kisumu. Broiler and layer farmers are highly specialized and almost all keep only one type of poultry. Indigenous chicken farmers, on the other hand, are less specialized with close to a fifth keeping some ducks. Other types of poultry found in Thika and Kisumu include turkeys, guinea fowls, quails, and pigeons, all of them mostly kept for aesthetic values rather than commercial values (Table 1). To further characterize poultry farming, poultry farmers are categorized according to the scale of production, income level and main occupation.

##### Scale of Poultry Production

Based on consultations with county agricultural staff, the low numbers of poultry kept in the study areas and for purposes of this study, poultry farmers were classified into three categories. Small, medium, and large-scale producers of indigenous chicken and ducks were considered to have between 1–50, 51–100 and above 100 birds, respectively. Layer and broiler production scale was categorized as small-scale with 1–200 birds, medium-scale with 201–500 birds and large scale with bird flocks above 500. Following this classification, the great majority of indigenous chicken farmers are small-scale and only a few are medium and large-scale producers. A majority of layer producers operate at small and medium-scale levels, while most duck farmers and broiler farmers are small-scale producers (Table 2).

**Table 2:** Scale of poultry production in Thika and Kisumu, 2016.

**Scale of production**	**Indigenous chicken (*n* = 254)**	**Ducks (*n* = 52)**	**Layers (*n* = 35)**	**Broilers (*n* = 33)**
Small	81.9	86.5	40	45.5
Medium	11	13.5	34.3	39.4
Large	7.1		25.7	15.2
Total	100.0	100.0	100.0	100.0

*Source: Author's survey, 2016*.

*All figures are percentages*.

#### Farmer-Suppliers Relationships

According to FGDs with farmers and county staff, Kenchic Limited is the predominant supplier of DOCs for broilers and layers. Apart from selling DOCs to individual farmers through their depots or agents, the company also contracts large-scale broiler farmers within Thika and surrounding counties. Under this out-grower arrangement, Kenchic Ltd., Nairobi, Kenya provides farmers with DOCs, feed, vaccines, and extension services, while farmers provide housing and management for broilers. Upon maturity, broilers from out-grower farmers are collected from the farms by the company and transported to the slaughterhouse in Thika, where they are slaughtered, processed, and packaged. These products are then sold to supermarkets and hotels. Kenchic Ltd., Nairobi, Kenya provides technical support to contracted farmers through their veterinary officers. In addition, the company trains aspiring poultry farmers at the headquarters in Nairobi. Other suppliers of DOCs include Muguku poultry farm and Kenya Agricultural and Livestock Research Organization (KALRO-Naivasha). The latter supplies improved indigenous chicken commonly referred to as KARI *Kienyeji*. Additionally, some DOCs available in both Kisumu and Thika are imported from Uganda. A few individual farmers, both in Thika and Kisumu, operate small-scale hatcheries for indigenous chicken, with capacities ranging between 500 and 700 eggs, weekly. Chicks of different ages from these sources are often sold to *agrovets* (shops selling agricultural inputs) and farmers. Prices of chicks vary with ages: day old chick cost Ksh. 80–90, 1-week chick -Ksh. 140, 2 weeks chick -Ksh. 170, 3 weeks chick -Ksh. 200, 1-month chick -Ksh. 240 (1 US Dollar was equivalent to Ksh. 103 at the time of the survey).

There were several feed producers in Thika, both large and small-scale. Most of the feed in Kisumu come from other counties, making feed expensive. During FGDs and KIIs in both cities, most farmers reported dissatisfaction with the quality of feed, vaccines, and drugs. Some feed companies in Thika have relationships with layer producers, whereby they provide credit in form of feed and later collect eggs, deducting the credit owed to them by producers. As explained by farmers during FGDs, this supplier relationship enables poultry producers to conduct their production smoothly because of the reduced financial burden, given that feed constitute the largest share of production costs, an observation corroborated by Okello et al. (24). However, farmers explained that acquiring credit in form of inputs could hold them captive suppliers, limiting their bargaining power.

#### Service Providers/Supporting Institutions

Service providers in the poultry value chain include extension and veterinary service providers, county livestock officers, credit service providers, NGOs that (such as SACDEP-Kenya and WEMIHS in Thika) train farmers or assist them with inputs and *agrovets* who stock poultry production inputs and offer livestock production advice. Extension and veterinary service providers operate as private practitioners or public county officers. FGDs with farmers revealed that most farmers are unaware of public extension, veterinary, and livestock production services which are offered free of charge, although demand driven. Instead, farmers mostly consult private veterinary officers, who (at a fee) provide advice to farmers on disease management and general poultry production. County agricultural and livestock officers also link producers to markets and service providers. Regarding training, only about a quarter of poultry farmers in the 2016 survey received training on poultry production during the previous year. The proportion of farmers who received training was statistically higher in Thika (30%) than Kisumu (19%). Similarly, poultry farming credit was received by 13 per cent of Thika farmers compared with only four per cent of Kisumu farmers.

Kisumu County with support from donors has been encouraging poultry production, especially in urban areas by providing grants in form of production inputs. The business starter package included free DOCs and feed. KIIs interviews with the County staff indicated that the aim was to replicate the poultry production micro-franchises in the County which will in turn create employment opportunities among women and youth.

#### Traders/Marketing

As shown in Table 3, there is an array of marketing channels for poultry and poultry products in Kisumu and Thika. A majority of broiler farmers sell their produce to hotels and restaurants and to brokers (middlemen).

**Table 3:** Poultry and poultry products marketing channels in Thika and Kisumu, 2016.

**Market channel**	**Broilers (*n* = 33)**	**Spent layers (*n* = 35)**	**Indigenous chicken (*n* = 254)**	**Eggs (*n* = 281)**
Broker	33.3	40	32.3	44.1
Retailer	3	5.7	3.1	12.8
Wholesaler	3	14.3		1.1
Supermarket				0.4
Hotel/restaurant	45.5	5.7	7.1	0.7
Processor			0.4	0.7
Direct sales	9.2	20	13.4	13.5
Contractor				0.7
Butchery	3		0.4	
Neighbors	3	14.3	43.3	26.0
Total	100	100	100	100

*All figures are percentages*.

*Source: Author's survey, 2016*.

Spent layers (spent layers are old layers which have finished production and are disposed-of for meat) are commonly sold to brokers and through direct sales at the market or sold to neighbors. Two fifths of indigenous chicken producers sell to neighbors while a third sell to brokers. The dominant marketing channel for eggs is brokers and neighbors (**Table 5**).

Hotels and restaurants often purchase on credit and later pay through checks which take a long time to process and mature, sometimes as long as 3 months. Most producers therefore prefer to sell to brokers or other market channels which pay in cash. Brokers, acting as middlemen, often purchase live birds from farmers and supply them to hotels and restaurants. Brokers offer the lowest price of Ksh. 233 per kg of broiler meat (chicken meat in Kenya is often sold with bones. Therefore, broiler meat implies broiler carcass including bones) while hotels offer Ksh. 283 per kg. Roast chicken meat traders, particularly in Kisumu also purchase live birds from farmers or slaughter them on-farm.

Because of low production volumes, stringent foods safety rules and production requirements, small-scale poultry farmers have a difficulty in accessing high end markets such as supermarkets, processing companies, and contractors, a characteristic of weak links (see Figure 1). Interviews with hotels' procurement managers revealed that in order to ensure food safety standards, hotels, and restaurants often purchase fresh chicken carcasses that have not been refrigerated, because it is easy to detect their freshness. In such an arrangement, it is the hotel that sets the price.

#### Governance and Institutional Framework

Thee understanding of the value chain governance structure and institutional relationships help in providing insights about power relations and how the rules of the game, written or unwritten influence relationships between actors. As mentioned, most poultry farmers in Thika and Kisumu operate as individual producers. Generally, most transactions are *ad hoc*, as no formal contracts exist between producers and traders. Only 22 farmers or seven per cent of the poultry farmers surveyed, produce poultry under contractual agreement with traders, hotels, and restaurants, input suppliers, or schools. However, almost all of these contracts are informal, with no written agreement, with the contractors' main roles being purchasers of produce (77%), inputs provision (27%) and provision of veterinary services (9%). In Thika, 13 per cent of farmers had contractual agreements compared to only three per cent in Kisumu. As disclosed by hotels procurement managers, they often require a steady supply of poultry products of high quality, conditions that are difficult to be met by small-scale urban poultry producers. Thus, the reason for low contractual agreement is partly because of low volumes produced by individual farmers and difficulties in meeting food safety standards.

The success of informal contracts is contingent on trust and social capital, as a result of repeated satisfactory transactions. This enhances reputation and reduces uncertainty, thereby reducing transaction costs (52). Integration with contractors or trading partners improves farmers' access to markets, credit (cash and inputs), extension services, market information and cash advances for up-front expenses (53, 54). Although supermarkets and by extension other forms of contracts often offer higher stable prices than conventional markets, delayed payments discourage most smallholders from accessing these market channels as was explained by farmers.

The urban poultry value chain governance under study mostly has a spot market structure. More than 60 per cent of farmers report that they negotiate output prices with buyers while 26 per cent decide on output price independently. Another four per cent report that buyers decide on price while six per cent indicate that price is often determined by forces of demand and supply (not presented). Perishability of poultry and poultry products and hence, the need to sell poultry immediately after attaining the market weight, also reinforces the existence of spot market governance structure. Farmers normally sell their flocks (broilers) immediately after attaining the market weight to reduce feed expenses. As a characteristic of spot market type of governance, most poultry farmers in this study produced their poultry without strict regulations on production and biosecurity. While having contractual agreements would reduce smallholders' risks of suffering losses, low produce volumes and food safety regulations limit their capacity to access niche markets like hotels and supermarkets.

Interestingly, even though most farmers report that they often negotiate output prices, FGDs reveal that most farmers believe that traders possess market power over them. Once chickens have matured or eggs are ready for sale, farmers have no option but to sell to brokers, who determine prices. Thus, they are being coerced to sell their produce under conditions determined by traders, for example, selling at the price set by traders without any price increment when the required weight is exceeded. Even though close to two thirds of poultry producers (65%) access market information from various sources, the most common source reported is traders (72%), who tend to give information that favor themselves (mostly giving the lowest price as the market price). Other sources of market information are fellow farmers (63%) and radio and television (4%).

#### Poultry Farmer Producer Groups

Producer organizations help in overcoming some challenges within the value chain. However, from this study, most poultry producers operate individually in small, medium, or large-scale. While the majority operate on an individual basis, some farmers organize themselves into poultry producer groups consisting of 10–15 members on average, including mostly women and youth but also a few men. However, it is only four per cent of poultry farmers in Thika and Kisumu who are members of producer groups. FGDs, KIIs, and interviews with poultry farmer producer groups revealed that lack of trust among producers is a major obstacle to the formation of functional producer groups. Likewise, in Nairobi, broiler farmers reported mistrust and lack of communication as a major obstacle to formation of farmer groups (31).

Poultry farmer groups operate as either registered (formal) or not registered (informal) entities, with some variations in the groups' arrangements. One type is poultry producer groups in which members produce poultry individually but market their produce as a group. In a different arrangement, poultry farmers contribute money for purchase of inputs and poultry production, group members provide labor, and they market their produce collectively. Given the many challenges of collective poultry production, the former type of poultry producer group arrangement is more common than the latter.

All the poultry producing groups (5 in Thika and 3 in Kisumu) interviewed were engaged in indigenous chicken farming, which they considered more profitable and easy to manage compared to layers or broilers. None of the groups interviewed engaged in full-time poultry production. They performed other income generating activities such as horticultural production, soap making, community sanitation, and entertainment businesses. These farmer groups often operate a revolving fund from which members can borrow money. Group membership also enables members to receive agricultural trainings from county agricultural and livestock officers, NGOs and input suppliers, who prefer training farmers organized in groups as opposed to individual farmers. Additionally, group marketing enables pooling of outputs from individually farmers, thus, increasing their marketable output and access to high value markets. Common challenges cited by poultry producer groups include lack of knowledge on poultry management and diseases, poor quality feed, and high cost of feed.

### Insights From Cluster Theory

#### Geographical Clustering of Producers and Input Manufacturers

Cluster theory provides both the demand and supply reasons for firms clustering in certain locations. Poultry and poultry product prices are generally higher in Thika than in Kisumu. Indigenous chicken and broiler meat fetch higher prices in Thika than in Kisumu. Furthermore, indigenous chicken eggs cost Ksh. 133 more per tray in Thika than Kisumu (Table 4). Thika is in close proximity to production inputs (DOCs and feed) and Nairobi, a major market for agricultural produce. Therefore, location within clusters, with respect to proximity to input and output markets appears to favor producers in Thika.

**Table 4:** Mean prices of poultry products/by-products in Thika and Kisumu, 2016.

**Enterprise**	**Type of product**	**Mean price (Ksh.)**	
		**Thika**	**Kisumu**
Layers	Eggs price/tray (30 eggs)	273 (14)	351 (127.2)^***^
	Spent birds	317 (43.7)	488 (112.5)^***^
	Manure (90 kg bag)	293 (110.8)	415 (171.7) ^***^
Indigenous chicken	Eggs price/tray (30 eggs)	520 (121.2)	387 (92.2) ^***^
	Spent birds	808 (289.9)	527 (207.3) ^***^
	Manure (90 kg bag)	250 (160.5)	280 (146.9)
Broilers	Broiler meat (Ksh/kg)	336 (45.5)	221 (98.8) ^***^
	Broiler manure (90 kg bag)	267 (103.1)	273 (186.0)

*Figures in brackets are standard deviations*.

*** *Significant at 1% (comparison of mean was tested using t-test)*.

*Source: Author's survey, 2016*.

In contrast, layers eggs fetch a higher price in Kisumu than in Thika (Table 4). This is because, Kiambu County, including Thika, is a nationally dominating egg producing region where egg prices are relatively lower (24), while Kisumu has less layer producers, which results in short supply of eggs and thereby higher prices. As a result of eggs shortage in Kisumu, some traders resort to importing from Uganda.

Firms often cluster in areas with adequate supply of inputs. Generally, poultry feed prices are lower in Thika than Kisumu, owing to a high concentration of feed companies in Thika and nearby Nairobi. Nearly all of the animal feed companies manufacture poultry feed, because poultry production is an important economic activity in the country (50). For layer production, the prices of all types of feed are significantly lower in Thika than Kisumu. Similarly, prices of all types of feed for indigenous chicken were significantly less expensive in Thika than Kisumu. For broilers, only finisher feed was more expensive in Kisumu than Thika. However, there were no significant differences in price of DOCs for layers, broilers and indigenous chicken across the two cities (Table 5). This could be attributed to the widespread distribution of Kenchic depots across the country and small-scale hatcheries producing indigenous chicken DOCs.

**Table 5:** Mean prices of poultry inputs in Thika and Kisumu, 2016.

**Enterprise**	**Type of product**	**Mean price (Ksh.)**	
		**Thika**	**Kisumu**
Layers	DOCs (per unit)	97 (11)	93 (16)
	Chick mash (per kg)	43 (4)	58 (10)^***^
	Growers mash (per kg)	39 (6)	48 (11)^***^
	Layers mash (per kg)	41 (5)	51 (10)^***^
Indigenous chicken	DOCs (per unit)	126 (61)	118 (65)
	Breeding stock (per unit)	491 (212)	381 (192) ^***^
	Chick mash (per kg)	46 (14)	62 (32) ^***^
	Growers mash (per kg)	44 (10)	52 (10) ^***^
	Layers mash (per kg)	45 (7)	50 (20)
	Other feed (per kg)	34 (12)	34 (12)
Broilers	DOCs (per unit)	63 (6)	63 (10)
	Starter (per kg)	60 (12)	63 (9)
	Finisher (per kg)	50 (1@)	61 (6)^**^

*Figures in brackets are standard deviations*.

** *Significant at 5%*,

*** *significant at 1% (comparison of mean was tested using t-test)*.

*Source: Author's survey, 2016*.

Table 6 presents the shares of costs in indigenous chicken production. Feed constitute about 70 and 76 per cent of costs in indigenous chicken production in Kisumu and Thika, respectively. DOCs account for about 20 per cent of total cost in the two cities. Other expenses incurred in poultry production are costs of heating and buying drugs.

**Table 6:** Shares of different costs in indigenous chicken farming in Kisumu and Thika, 2016.

	**Share of cost (%)**		
**Item**	**Kisumu**	**Thika**	**Whole sample**
Feed	70	76	73
DOCs	18	22	19
Drugs	10	1	7
Heat	2	1	1

*Source: Author's computation, 2016*.

The gender of the poultry owner (indigenous chicken) and gross margin, an indicator of business profitability, are presented in Table 7. Nearly half of the poultry enterprises were co-owned by both males and females in the household (mainly husband and wife). However, female ownership of poultry enterprises was higher (32%) than male ownership (20%). The most profitable indigenous chicken production venture was among the co-owned ventures (Ksh. 894/bird) followed by ventures owned by females (Ksh. 693/bird).

**Table 7:** Gender of owner of indigenous chicken venture and profitability.

**Gender**	**Proportion (%) (*n* = 149)**	**Gross margin (Ksh./bird)**
Female	31.9	598
Male	19.7	693
Co-owned by male and female	48.4	894

*Computation of gross margin and proportion is for venture having 20 or more birds*.

*Source: Author's computation, 2016*.

**Figure 2:**
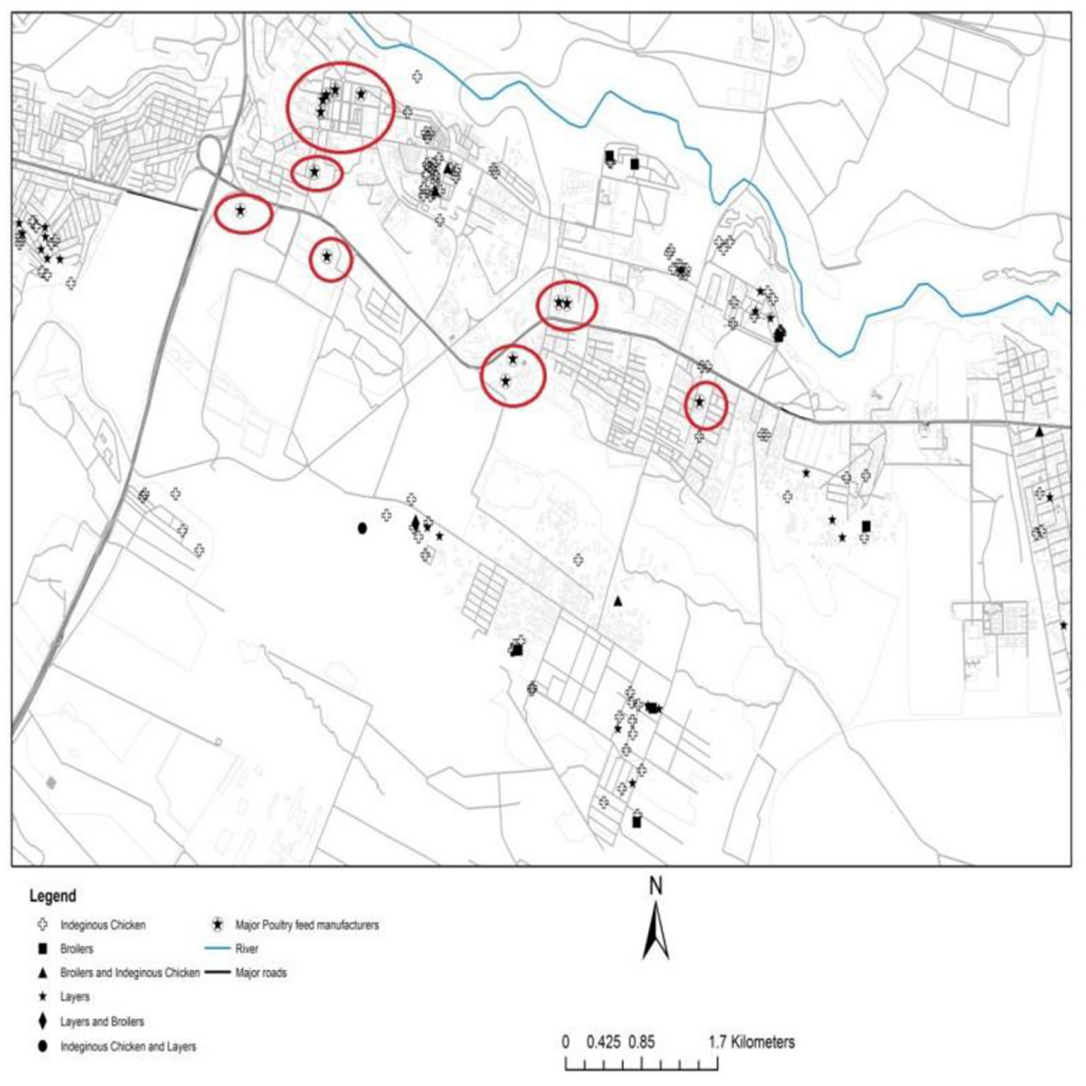
Map of Thika showing location of poultry producers and input markets, 2016.

Figure 2 shows the location of major feed manufacturers and location of producers in Thika. There is a high concentration of feed manufacturers in Thika (marked in red circles). These are only the registered companies, implying the number of feed millers in Thika could be much higher than the number reported here. The high number of feed manufacturers and proximity to producers in Thika could be the reason why feed prices are significantly lower in Thika than Kisumu. The competition between relatively many feed millers in Thika forces prices downwards compared to Kisumu. While some of the poultry and poultry products produced in Thika are consumed within Thika, a significant share is sold in Nairobi or to other parts of the country, which translates to generally higher outputs prices for Thika farmers than Kisumu farmers as presented earlier in Table 4.

**Figure 3:**
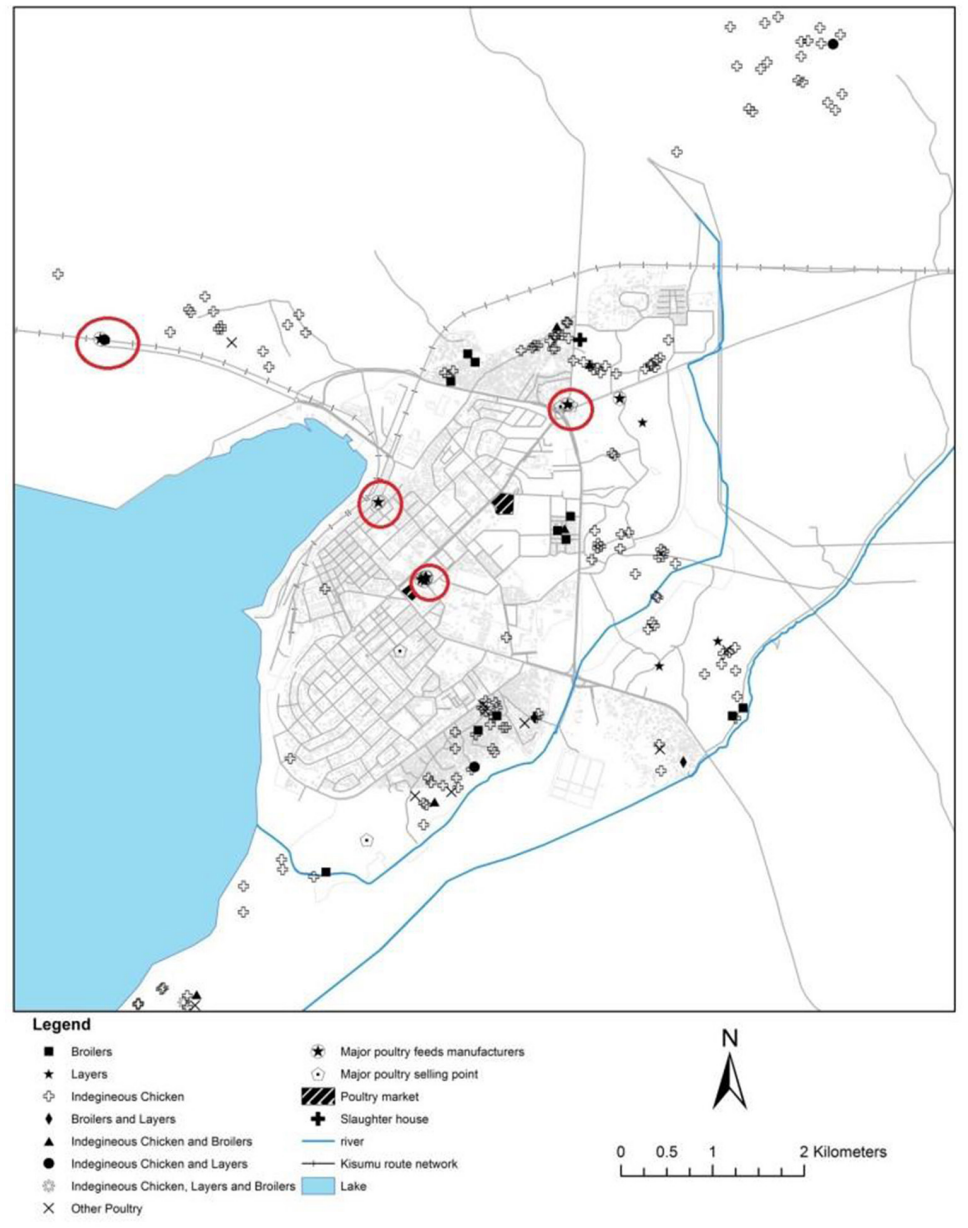
Map of Kisumu showing location of poultry producers and input and output markets, 2016.

In contrast, there are relatively fewer feed companies in Kisumu than Thika as shown in Figure 3. In Kisumu, most producers are located far from the feed companies. The feed companies are also sparsely distributed unlike in Thika. The cost of transportation to local *agrovets* increases the final feed price paid by producers. Furthermore, the less stiff competition among feed manufacturers in Kisumu could partly explain the high feed prices in Kisumu. There are specific poultry selling points in Kisumu, particularly roadside sellers along the roads and outside night clubs who sell ready-to-eat chicken. In addition, there is a slaughterhouse in Kisumu, though far from most producers.

#### Local Level Relationships, Smallholders' Strategies, and Competition

Through cluster theory and analysis of local level factors such as interactions between actors, cooperation and competition help define the operation of a value chain. Apart from marketing channels presented in Table 5, FGDs and KIIs with farmers revealed that young poultry producers market their poultry and poultry products through the internet and social media platforms such as OLX, Facebook, and Whatsapp. Others advertise through posters placed in strategic locations such as in *agrovets* and outside county agricultural and livestock offices. Most farmers also sold their produce through referrals from their colleagues. Surprisingly, a majority of farmers reported that they share market information (78%), meaning that they also collude in setting output prices. These upgrading mechanisms help smallholders to remain competitive in poultry production. Because of the stiff competition, traders dealing with offal, i.e., chicken legs, intestines, and heads sometimes slaughter broilers for traders dealing with broiler meat in order to ensure access to all the offal. Through this strategy, all the edible poultry by-products are taken by those who slaughter on behalf of the traders or farmers. Most dealers in chicken offal add food color and fry the parts as a marketing strategy. For the roast meat traders, especially in Kisumu, they often serve chicken with complementary *ugali* (a thick paste made from maize meal) and *kachumbari* (a salad composed of chopped tomatoes and onions).

The study reveals that local level relationships among farmers enhances knowledge spill over. For instance, 38 per cent of respondents reported that they acquired poultry production knowledge from friends and relatives while 39 per cent learned the practice from their parents. Another 17 and 9 per cent acquired the knowledge from input providers and mass media/internet, respectively.

Suppliers of DOCs mostly provide technical support to farmers as a means of attracting customers. For example, they offer advice on poultry production and management on issues such as feeding and vaccination. Additionally, others offer credit, through inputs while some like feed manufacturers also engage in selling chicken cages and purchasing of eggs from farmers.

Traders dealing with fried or roast chicken also use a number of marketing strategies. For instance, they sell chicken in small parts which are affordable to most people. Additionally, they diversify their incomes by selling other meat products such as roast beef.

This study found that there are cases of poultry theft especially in high density low-income areas. Poultry theft is more prevalent in Kisumu than Thika and was reported by 16 and 6 per cent of poultry farmers in the two cities, respectively. The stolen poultry is later sold or consumed. Another illegal activity is slaughtering of poultry intended for sale at home/on-farm or in hotels without inspection by public health officers. As reported earlier, more than three quarters of broiler farmers slaughter their chickens without inspection. Additionally, most hotels and restaurants slaughter chicken without inspection. For instance, at a certain slaughterhouse (name and city withheld), the county meat inspectors reported that only one hotel slaughters its chicken at the slaughterhouse. During interviews, hotel procurement managers of two well-reputed hotels in that city reported that poultry slaughtered at the hotel is usually inspected by county officials, a claim denied by the County meat inspectors. Such cases of neglect are in violation of existing regulations as stipulated by the meat control act that requires all poultry intended for sale to be inspected (55). The illegal slaughtering is a means of avoiding production costs in paying for meat inspection and chicken transportation to the slaughterhouses. For instance, at time of the study, at Mamboleo slaughterhouse in Kisumu, the County government charged Ksh. 10/bird while the meat inspector was paid Ksh. 2 per bird.

To reduce food safety risks, most hotels source their poultry from specific farmers, usually 3–5 farmers whom they have informal contracts with. However, during scarcity, hotels source poultry from any other farmer with poultry. It is also common for the hotels to purchase poultry from brokers who in turn buy poultry from diverse farmers. This saves the hotels the task of moving from farmer to farmer in search of poultry.

FGDs with farmers also indicated that most farmers do not observe the waiting period of selling poultry products after drugs administration. This implies that those who use excessive antibiotics for disease prevention and growth promotion and refrain from adhering to the withdrawal period before the produce is sold or consumed, may cause antibiotic resistance among consumers of these products. Some farmers claimed to use Anti-Retro Virus drugs (ARVs) to fatten their broilers, while others, including traders, sell sick birds to unsuspecting consumers to avoid losses.

Another common illegal activity is the production and sale of poor quality feed. This is as a result of the rising number of animal feed companies in the country, some with doubtful operation licenses. According to FGDs with farmers, the companies usually attract customers by initially selling good quality feed. However, once a substantial market base has been established, feed quality deteriorates, leaving farmers at a risk of incurring losses because of delayed maturity in birds and low productivity. Furthermore, despite the existence of a regulation on transportation of livestock, most poultry farmers and traders transport live birds in public service vehicles without movement permits. Others use motorbikes and bicycles, which is prohibited.

This section has presented the impact of local level interaction, strategies, and competition in the urban poultry value chain. To remain competitive, producers must innovate or upgrade their production activities and marketing. However, to some producers, the means of upgrading are expensive thus they deploy illegal “upgrading” mechanisms to remain in business. While it is required that all meat intended for marketing should be inspected, most producers slaughter on-farm without inspection, which would attract extra expenses. Instead of purchasing high quality feed, which comes at an extra cost, some producers use some risky means to fatten their poultry by using human ARVs and growth hormones. Similarly, some feed millers do not adhere to the feed quality standards and use inappropriate ingredient ratios to maintain their competitiveness in the sector. The competition also enables smallholders to use alternative marketing channels such as the internet, cooperation among themselves and through referrals.

## Discussion

Small-scale poultry production and trade, which can be considered as micro-enterprises, offer employment opportunities to the actors. It has the potential to contribute to the achievement of the Sustainable Development Goals of improved food security and alleviating poverty (20).

The contextualization of the poultry value chain in medium-sized cities offers useful insights on how the value chain operates unlike in rural areas where there are relatively large tracts of land for extensive agriculture, the small urban agricultural land does not allow for large scale production. This is shown by the relatively few numbers of poultry kept by urban farmers compared to those in rural areas (24). The value chains are also relatively shorter because most of the products are consumed locally, for instance there is no need for cold storage by farmers. However, in an effort to remain competitive, a small share of producers and traders engage in illegal “upgrading” activities such as illegal slaughtering of poultry without inspection, theft and manufacture of adulterated feed.

The poultry value chain in the two cities surveyed is dominated by a spot market form of governance with informal arm's length transactions, in which trading partners are independent. There are limited contractual arrangements between farmers, input suppliers and traders. The commodity value chain's governance often benefits farmers toward the hierarchy form of governance as earlier described (34, 56). As transactions become more complex and asset specificity increases, so does the governance structure. In these higher governance forms, the purchasing firm exerts stringent requirements to suppliers (farmers), for example on food safety, production, and consistency in supply and quality in order to meet the market requirements (23, 33, 57). For instance, broiler out-grower farmers have to observe production requirements and biosecurity levels dictated by the contracting firm (31), while in a spot market, as shown in this study, farmers generally conduct their production without strict regulation from the buyers.

Studying the activities of value chain actors from a cluster theory perspective also yields useful insights. Cluster theory emphasize on the advantages of clustering and importance of innovation and upgrading in the ever increasingly competitive market (26). Among Thika poultry farmers, being located in a cluster of feed producing companies, commercial livestock farmers, commercial crop production, and other related industries translates to advantages in terms of availability of specialized inputs (feed) at relatively cheaper prices than in Kisumu. This makes farmers in Thika more competitive. A location within a cluster reduces input costs while, at the same time, it lowers transaction costs and eases the flow of information. Additionally, close proximity to Nairobi which offers a wide market base for agricultural products means that farmers in Thika enjoy better output prices than Kisumu farmers. The reduced distance in transporting inputs to the farms and output to the market makes them more competitive. This analysis has demonstrated that two of the advantages of firms' agglomeration are present in Thika, i.e., ease of accessing specialized inputs and a demanding market (26, 38).

Urban poultry farmers innovate or upgrade in various ways to maintain their competitiveness. Young farmers employ the social media and online platforms to market their produce. Others use effective advertising tools such as placing advertisement at strategic locations like in *agrovets* or at county agricultural offices. Others, through their social networks market their produce through referrals. However, some producers and traders “upgrade” through illegal means such as slaughtering of poultry without inspection, theft and sale of adulterated feed. All these aim at reducing production and marketing costs.

There are only a few studies, all focusing on developed countries that have focussed on illegalities in agricultural value chains (58–60). Otherwise, food crime rarely features in agricultural or food policy literature (60). This is despite the growing number of cases of illegality in agricultural activities reported, some of which pose food safety and health risks to consumers, especially in the less developed countries. This study has shown that in the wake of stiff competition, some individuals/firms in the agricultural and food industry engage in illegal activities to be competitive.

## Conclusion

This study aimed at understanding the operation of urban poultry value chain in the context of medium-sized cities in Kenya. This was facilitated by combining value chain approach and cluster theory. The study has demonstrated that small-scale poultry production continues to be a viable and profitable enterprise in urban settings in Kenya, especially among women. In Kenya, the demand for poultry is projected to triple between 2000 and 2030, presenting opportunities to urban poultry producers. This will be occasioned by increasing health consciousness and preference for white meat and rising incomes. Urban poultry farming supports an array of actors including, producers, input and output traders. Spot market is the most common type of value chain governance in the surveyed cities.

As shown in this study, farmers selling to high value markets (such as to hotels and restaurants) get better prices than those selling to brokers. Formation of producer groups might help smallholders in upgrading these markets by pooling their output and putting pressure to group members to adhere to food safety requirements. Farmers also expressed the need for acquiring knowledge about poultry production and poultry diseases through extension and veterinary services which could help them improve their position in the poultry value chain.

Poultry theft, slaughtering of chicken without inspection, disregarding drugs withdrawal period and manufacturing of substandard poultry feed are some of the illegalities in the urban poultry value chain. Farmers should be educated through agricultural extension on the importance of adhering to drugs withdrawal periods and only use recommended drugs at appropriate doses. The county governments should make it mandatory that all hotels slaughter poultry in slaughterhouses under inspection by meat inspectors to ensure food safety. To curb the problem of poor quality inputs, Kenya Bureau of Standards (KEBS) should ensure that only certified individuals and companies produce poultry production inputs, particularly feed.

In terms of practice, urban poultry production could be promoted by the local authorities as an income diversifying enterprise (61). However, the promoters should be aware of the “upgrading” mechanisms that pose food safety risks to consumers of poultry products and should therefore, educate producers on the same.

At the time of writing this paper, there was no any pandemic or situation that affected the supply of inputs and marketing of output. However, COVID-19 pandemic has greatly affected the operation of many businesses including poultry production. Restrictions aimed at curbing COVID-19 such as curfews and closure of public meeting places and hotels imply that the demand for poultry and poultry products has slightly reduced. Interviews conducted with county staff in June 2021 show that some producers could no longer operate and thus had to close down. The spot market identified in the two cities implies that there is low asset specificity among most producers. This means there is ease of exit or entry into the industry. However, as the situation is improving, it is expected that slowly, poultry production will return to normal.

## Limitation of the Study

Computation of the value added is an important component of value chain analysis. However, the value added was not computed due to difficulties in acquiring accurate information from companies, traders and hotels to enable computation of value added.

## Data Availability Statement

The raw data supporting the conclusions of this article will be made available by the authors, without undue reservation.

## Ethics Statement

Ethical review and approval was not required for the study on human participants in accordance with the local legislation and institutional requirements. Written informed consent for participation was not required for this study in accordance with the national legislation and the institutional requirements.

## Author Contributions

SO designed the study, conducted data collection and analysis, and manuscript development.

## Funding

This work was supported by the Swedish Research Council for Environment, Agricultural Sciences and Spatial Planning (FORMAS) (grant number: 225-2012-609) and the Swedish International Development Cooperation Agency (SIDA) (grant number: SWE-2011-028).

## Conflict of Interest

The author declares that the research was conducted in the absence of any commercial or financial relationships that could be construed as a potential conflict of interest.

## Publisher's Note

All claims expressed in this article are solely those of the authors and do not necessarily represent those of their affiliated organizations, or those of the publisher, the editors and the reviewers. Any product that may be evaluated in this article, or claim that may be made by its manufacturer, is not guaranteed or endorsed by the publisher.
